# Characterization of the Complete Mitochondrial Genome Sequences of Three Croakers (Perciformes, Sciaenidae) and Novel Insights into the Phylogenetics

**DOI:** 10.3390/ijms19061741

**Published:** 2018-06-12

**Authors:** Huirong Yang, Jun Xia, Jia-en Zhang, Jinzeng Yang, Huihong Zhao, Qing Wang, Jijia Sun, Huayi Xue, Yuanyuan Wu, Jiehu Chen, Jingchuan Huang, Li Liu

**Affiliations:** 1 College of Marine Sciences, South China Agricultural University, Guangzhou 510640, China; hry@scau.edu.cn (H.Y.); zhaohh@scau.edu.cn (H.Z.); wangqing@scau.edu.cn (Q.W.); jjsun@scau.edu.cn (J.S.); huayisher@sina.com (H.X.); hg0hgoo7@163.com (Y.W.); 2 Department of Human Nutrition, Food and Animal Sciences, University of Hawaii at Manoa, Honolulu, HI 96822, USA; jinzeng@hawaii.edu; 3 Xinjiang Acadamy of Animal Sciences, Institute of Veterinary Medicine (Research Center of Animal Clinical), Urumqi 830000, China; xiajun2004263@163.com; 4 Guangdong Engineering Research Center for Modern Eco-Agriculture and Circular Agriculture, Guangzhou 510642, China; jeanzh@scau.edu.cn; 5 Science Corporation of Gene, Guangzhou 510000, China; chenjiehu@scgene.com (J.C.); huangjingchuan@scgene.com (J.H.)

**Keywords:** *Nibea coibor*, *Protonibea diacanthus*, *Argyrosomus amoyensis*, Sciaenidae, mitochondrial genome, characterization, phylogenetic analysis

## Abstract

The three croakers (*Nibea coibor*, *Protonibea diacanthus* and *Argyrosomus amoyensis*, Perciformes, Sciaenidae) are important commercial species inhabiting the Eastern Indian Ocean and Western Pacific. Molecular data employed in previous research on phylogenetic reconstruction have not been adequate and complete, and systematic and comprehensive phylogenetic relationships for these fish are unresolved. We sequenced the complete mitochondrial genomes of the three croakers using next-generation sequencing for the first time. We analyzed the composition and phylogenies between 19 species in the family Sciaenidae using the mitochondrial protein coding sequences of 204 species in the Series Eupercaria. We present the characterization of the complete mitochondrial genome sequences of the three croakers. Gene arrangement and distribution of the three croakers are canonically identical and consistent with other vertebrates. We found that the family Sciaenidae is an independent branch that is isolated from the order Perciformes and does not belong to any extant classification. Therefore, this family is expected to belong to a new classification at the order level and needs further analysis. The evolution of Sciaenidae has lagged far behind the Perciformes differentiation. This study presents a novel insight into the phylogenetics of the family Sciaenidae from the order Perciformes and facilitates additional studies on the evolution and phylogeny of Series Eupercaria.

## 1. Introduction

*Nibea coibor* (light colored croaker), *Protonibea diacanthus* (black spotted croaker), and *Argyrosomus amoyensis* (Amoy croaker) are tropical marine demersal fish mainly inhabiting the eastern Indian Ocean and the western Pacific, including the South China Sea (http://www.fishbase.org/search.php) [1,2,3]. They feed on crustaceans and small fish and have high commercial value due to their nutritional and medicinal properties [4,5,6,7].

Mitochondrial DNA (mtDNA) is an important model system for studying molecular evolution, phylogeny, and genome structure [8,9]. The size of the mitochondrial genome is approximately 15–20 kb and contains 13 protein-coding genes (PCGs), two ribosomal RNAs genes (*12S rRNA* gene and *16S rRNA* gene), 22 transfer RNA (*tRNA*) genes, and a major non-coding region that contains the initial sites for mtDNA replication, and RNA transcription [10]. Fish mitochondrial genomes have a similar organization to other vertebrates [11]. Because of a constant gene content, multiple copy status in a cell, and a lack of recombination and paralogues, this is of particular interest as the mitochondrial genome is a simpler system than the nuclear genome for studying the molecular dynamics and mechanisms of rearrangements that underlie variations in the genome. Other features of mtDNA are its small size, cellular abundance, maternal inheritance, compact gene arrangement, and high rate of evolution. Therefore, these make the mitochondrial genome a particularly attractive tool for studying evolutionary biology [12,13].

Thus, mtDNA is generally considered a good molecular marker for phylogenetic analyses among fish taxa. However, short mitochondrial gene fragments exhibit limitations in resolving complicated phylogenetic relationships in many fish lineages [14]. The additional informative sites from longer DNA sequences (e.g., mitochondrial genomes) allow these deeper branches and higher-level relationships to be more fully resolved [15]. Hence, the mitochondrial genomes provided in this study may help resolve the evolutionary relationships of the family Sciaenidae and the Series Eupercaria and lead to taxonomic revisions.

There are only 9 studies on *N. coibor*, *P. diacanthus*, and *A. amoyensis* [16,17,18,19,20,21,22,23] involving active material extraction, nosography, and mitochondrial phylogenetic and microsatellite variation. There have been some studies concerning mitochondrial genome in the genera *Nibea*, *Protonibea*, and *Argyrosomu*, the family Sciaenidae and order Perciformes. However, investigations of phylogenetic relationships have been limited because they are based on partial mitochondrial sequence fragments, particularly *COXI* and the *16S rRNA* gene, since few mitochondrial genomes have been sequenced [24]. Only a portion of the mitochondrial genomes is used for the taxonomic classifications, and none have yet been comprehensively described [25,26,27,28,29,30]. Systematic and comprehensive phylogenetic relationships within the Series Eupercaria have not been resolved and the molecular data employed in previous research on phylogenetic reconstruction is incomplete. Therefore, we sequenced the complete mitochondrial genomes of three croakers using a next-generation sequencing (NGS) strategy in this study.

Firstly, our interest in this study was to compare the complete mitochondrial genome sequences of these fish which were sequenced in our study for insights into their characteristics, including their genome organization and composition, gene content, functional regions and the evolutionary dynamics and molecular mechanisms that have shaped them. Beyond this, these completely sequenced mitochondrial genomes provide a broad and useful molecular resource.

Secondly, the family Sciaenidae is a diverse and commercially important family, one of the largest within the Perciforme, comprising 68 genera and about 311 species [31]. We took a detailed view of the composition and phylogeny in 19 species including representatives from 12 of the entire16 genera in family Sciaenidae, based on the mitochondrial genomes presented in GenBank.

Thirdly, Series Eupercaria (=Percomorpharia in previous versions of the classification) possesses more than 6000 species arranged in 161 families and at least 17 orders [32], and is by far the largest series of Percomorphs. Some of the most diverse orders of fish are included in this group. Previous molecular studies obtained monophyletic groups with a combination of taxa assigned to Eupercaria, but including a far more limited sampling [33,34,35]. Although most family-level and ordinal groups within this series receive high nodal support, interrelationships among them are largely unresolved [36]. As currently circumscribed, the order Perciformes is the largest group within Eupercaria and has been traditionally regarded as a “taxonomic waste basket” [31]. Therefore, we preformed the phylogenetic analyses and divergence times based on the concatenated alignment of 13 PCGs amino acid sequences of 204 species of Series Eupercaria in this study. 

To sum up, according to the above description and situation, not only does this article provide new insight into novel mitochondrial genome organization among the family Sciaenidae, but it also proposes a monophyletic definition based on robust molecular analyses and a revised taxonomic circumscription of Perciformes and Series Eupercaria. All information reported in this article may facilitate further investigation of the population genetic structures, phylogenetic studies and molecular evolution of Sciaenidae. This work also provides important new molecular resources for the species identification, fishery management, and conservation biology with respect to Sciaenidae.

## 2. Results and Discussion

### 2.1. Genome Organization and Composition

**Figure 1:**
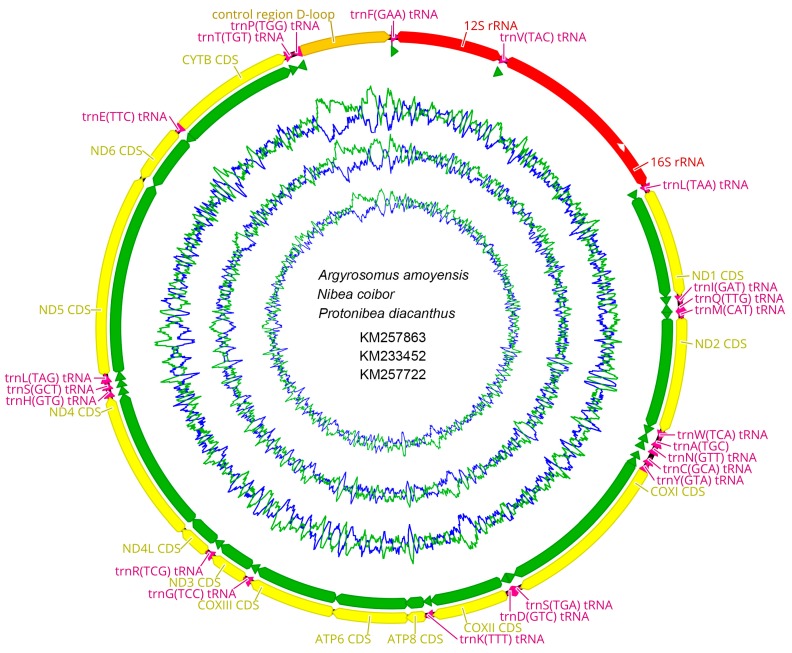
Gene map of the complete mitochondrial genomes for *A. amoyensis* (GenBank accession NO. KM257863), *N. coibor* (KM233452), and *P. diacanthus* (KM257722). The larger ring indicates gene arrangement and distribution, the genes on the outer circle are encoded by the H-strand, and those on the inner circle are encoded by the L-strand. The smaller ring indicates the GC content. *ND1-6*: NADH dehydrogenase subunits 1–6; *COXI-III*: cytochrome c oxidase subunits 1–3; *ATP6* and *ATP8*: ATPase subunits 6 and 8; *Cytb*: cytochrome b, D-loop: control region.

The complete mitochondrial genomes of *A. amoyensis* (GenBank accession No. KM257863), *N. coibor* (KM233452), and *P. diacanthus* (KM257722) were 16,490 bp, 16,502 bp, and 16,535 bp in length, respectively. They contained the canonical 13 PCGs, 2 ribosomal RNA genes (12S and 16S), 22 *tRNA* genes, and a control region (D-loop). Like many teleost fish, most of these were encoded on the heavy strand (H-strand) except NADH dehydrogenase subunit 6 (*ND6*) and 8 *tRNA* genes for Gln, Ala, Asn, Cys, Tyr, Ser, Glu, and Pro on the light strand (L-strand). The gene GC content varied from 34.29 to 57.35% and the 16S and *12S rRNA* genes were located between *tRNA-Leu* and *tRNA-Phe* genes and separated by *tRNA-Val* (Figure 1).

Gene arrangement and distribution of the three croakers were canonically identical and consistent with other vertebrates [37,38,39,40,41,42,43,44,45] but much different from the Mollusca [46]. However, the commonly discovered *tRNA* cluster Glu-Thr-Pro was not present in the three studied croakers or the other 9 studied teleosts [47], which covered Perciformes, Cypriniformes, and Siluriformes [38,39,40,41,42,43,44,45]. The cluster *tRNA-Glu*-*cyb-tRNA-Thr-Pro* was available instead [48].

The mitogenomes of *A. amoyensis*, *N. coibor*, and *P. diacanthus* were considerably shorter than those of *Johnius belangerii* and *Johnius grypotus* but were consistent with the average of other Sciaenidae species (Table 1). Intergenic spacers in *N. coibor* (13), *P. diacanthus* (14), and *A. amoyensis* (11) involving 90, 130, and 67 bp, respectively, indicated a greater diversity in the locations and intergenic nucleotides compared to the overlaps. A *tRNA-Phe*―*12S rRNA* intergenic spacer (45 bp) of *P. diacanthus* was the largest and distinctly different with those of *A. amoyensis* (0 bp) and *N. coibor* (0 bp), which considerably contributed to the longest complete mitogenome of *P. diacanthus* among the three croakers in this study (Table 2). The 34–36 bp intergenic spacers located in a common cluster of five *tRNA* genes between *tRNA-Asn* and *tRNA-Cys* were secondary and almost identical between the three croakers (WANCY region, Table 2).

The overlaps of the mitochondrial genes were 20 bp for *N. coibor*, 29 bp for *P. diacanthus*, and 29 bp for *A. amoyensis* and occurred in 7 different locations at 1–10 bp, resulting in a compact mitochondrial genomic structure. Most overlaps were detected in *tRNA-Ile―tRNA-Gln, tRNA-Gln―tRNA-Met, COXI―tRNA-Ser, ND4L―ND4, and ND5―ND6*. Generally, we allowed gene overlaps between adjacent genes but seldom between PCGs and *tRNAs*. When a full termination codon (TAA or TAG) caused an overlap between a protein-coding gene and a *tRNA*, we annotated this gene using an incomplete termination codon T/TA rather than as an overlap. We annotated full termination codons, however, in overlapping PCGs (Table 2).

### 2.2. Nucleotide Composition

The overall nucleotide compositions of the H-strand of the three croakers in descending order were 31.24% C, 27.13% A, 25.28% T, and 16.35% G, with an A+T content of 52.41% (varying between 52.07–52.88%), indicating significant strand asymmetry or strand-specific bias. This is commonly observed in most vertebrates [29]. The GC-/AT-skews analysis indicated that the H-strand possessed an overrepresentation of C and A, and consequently contained a lower number of G and T bases. Strand asymmetry due to nucleotide composition was also reflected in the codon usage of genes oriented in opposite directions. Genes encoded on H-strand showed a clear preference for C in codon wobble position, whereas G or T ending codons were overrepresented in the genes on the L-strand. The underlying mechanism responsible for the strand bias has generally been interpreted as evidence of an asymmetrical directional mutation pressure. This is associated with replication processes as one strand remains transiently in a single-stranded state, making it more vulnerable to DNA damage [49,50].

The highest A+T content was detected in the control region (64.08%) of *P. diacanthus* consistent with previous reports on other teleosts, but this occurred in *tRNA-His* (65.22%) in *N. coibor* and *tRNA-Pro* (65.71%) in *A. amoyensis* [51,52].

We compared the mitochondrial genomic nucleotide compositions of 19 Sciaenidae species. Strand asymmetry due to nucleotide composition could be described by AT and GC skew values, and the AT skews among these 19 species near zero, while their GC skews were all negative, except for *J. grypotus* and *J. belangerii*, which were the converse (Table 1). The A content of most species was only slightly higher than T, whereas C was considerably more prevalent than G. Such skews towards a particular nucleotide were attributed to differential mutational pressures imposed on the L- and H-strands resulting from the asymmetric replication of mtDNA. It was apparent that A and C were more prevalent across the entire mitogenomes of most of these Sciaenidae species, similar to previous observations of a bias against the use of G [47,53].

The diversity of the PCGs showed a different situation. The AT skews of 16 of these 19 species were barely less than zero and was the converse for the entire genome. The GC skews were all negative except for *J. grypotus* and *J. belangerii*, which were consistent with the entire genome. The T content of most species was only slightly higher than A, whereas C was considerably more prevalent than G in the PCGs. It was apparent that T and C were more prevalent in the PCGs of most of the Sciaenidae species. This was again similar to previous observations of a bias against the use of G [47,53]. Notably, *J. grypotus* and *J. belangerii* had the longest complete mitochondrial genomes and possessed unusual nucleotide skews (Table 1). The reasons for this are unclear at present.

**Figure 2:**
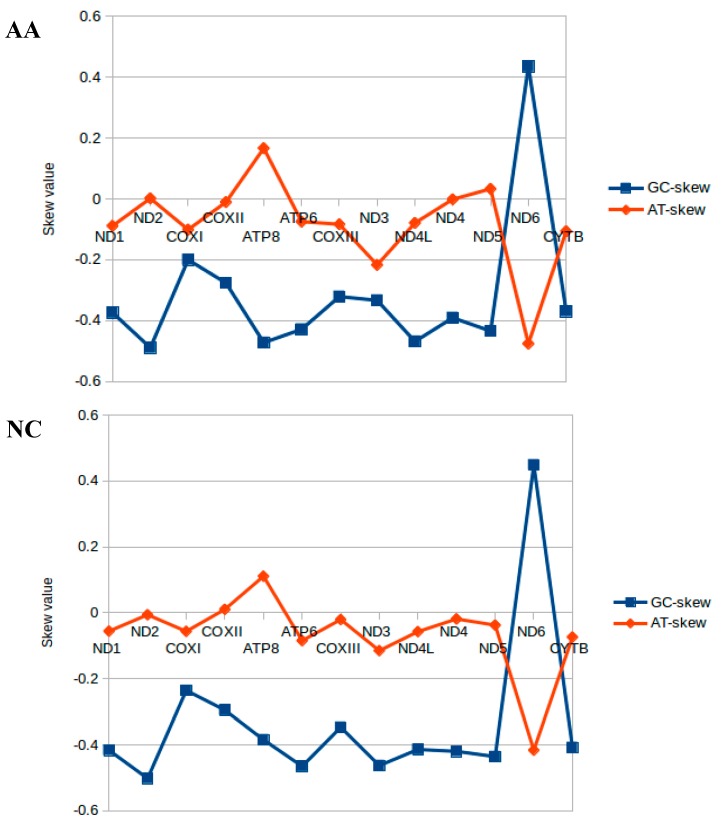

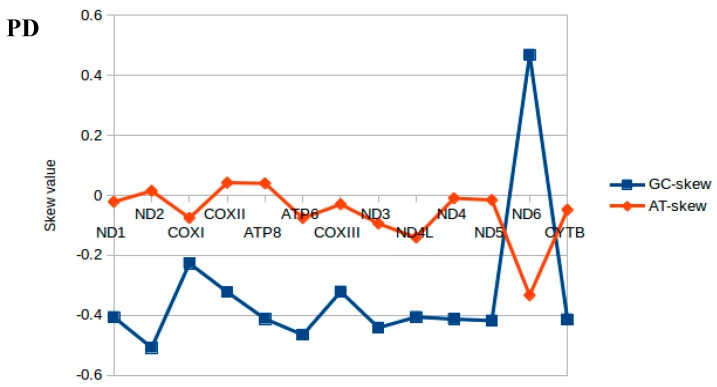
Graphical illustration showing the AT- and GC-skew in the PCGs of the mitochondrial genome of *A. amoyensis* (AA), *N. coibor* (NC), and *P. diacanthus* (PD).

The AT skews in the 13 PCGs of the three croakers were waving near the zero, and the majority of them were negative. The GC skews were all negative except for the *ND6* gene. This situation was consistent with the other 16 species, but, interestingly, the AT skews for *ND6* ranged from −0.4757 to −0.333 and GC skews ranging from 0.4353 to 0.4684 (Figure 2). These values were unusual but similar to other observations of strand asymmetry [47,53].

### 2.3. Protein-Coding Genes

The 13 PCGs in the mitogenomes of the three croakers were similar to those of other vertebrates. These included 3 cytochrome c oxidase complexes (*COX I–III*), 7 NADH ubiquinone oxidoreductase complex (*ND1-6*, *ND4L*), a cytochrome b oxidoreductase complex (*Cyt b*), and 2 ATP synthases (*ATP6* and *ATP8*). The coding regions ranged in size from 168 (*ATP8*) to 1839 bp (*ND5*) comprising 11,404–11,435 bp accounting for approximately 69% of the entire mitogenomes. The majority of these genes had similar lengths except for *ATP6* and *ND5* that possessed 27 and 15 bp gaps, respectively. All protein-coding genes were located on the H strand except for *ND6*.

Initiation and termination codons were determined based on alignments with the corresponding genes and proteins of other Sciaenidae fish. The mitogenomes of the three croakers exhibited a canonical genetic code shared by all vertebrates. An orthodox initiation codon methionine (ATG) was used for most of the PCGs, while GTG was utilized in *ND1* of *N. coibor* and in *ATP6* of *P. diacanthus*, ATA in *ATP6* of *N. coibor*, and ATC in *ND5* of *P. diacanthus.* These were also canonical mitochondrial initiation codons for vertebrate mitogenomes [54].

The termination codons AGA, T, TA, TAA, and TAG were present in all three species, and T and TAA were the most prevalent. Incomplete termination codons using either TA or T would be completed as a TAA via post-transcriptional polyadenylation. A diverse pattern of codon usage within stop codons seems to be a common tendency in fish mitogenomes. Since the three croakers were AT-rich organisms, we expected that A or T ending codons would predominate. The codon usage patterns in the 13 PCGs of the three croaker mitogenomes was similar to bony fish (Osteichthyes) [55].

**Figure 3:**
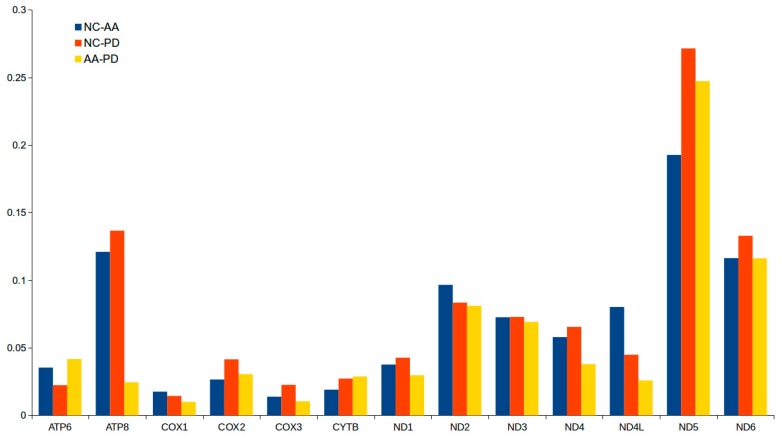
Evolutionary rates of the mitochondrial genome of *A. amoyensis* (AA), *N. coibor* (NC), and *P. diacanthus* (PD). The ratio means the rate of non-synonymous substitutions to the rate of synonymous substitutions (Ka/Ks) for each PCG.

In all 13 PCGs of the three croakers, the average non-synonymous/synonymous mutation ratio (Ka/Ks) was 0.0671 and varied from 0.0100 (*COXI* between AA-PD) to 0.2714 (*ND5* between the NC-PD) (Table S2, Figure 3). Two non-adaptive forces, random genetic drift, and mutation pressure define the fundamental features of genome evolutional though functional constraints imposed burdens on mutation events [56]. Therefore, the mutation-associated disadvantages were difficult to establish under purifying selection. The selection processes maintained long-term stability of the biological structure. Our Ka/Ks ratio indicated that the functional genes evolved under strong purifying selection, which meant natural selection against deleterious mutations with negative selective coefficients [57]. The selection pressures differed among the genes and were likely to evolve in different ways [56]. Additionally, the *ND5* genes had the highest Ka/Ks values, indicating that the selection pressures were strand-independent.

**Figure 4:**
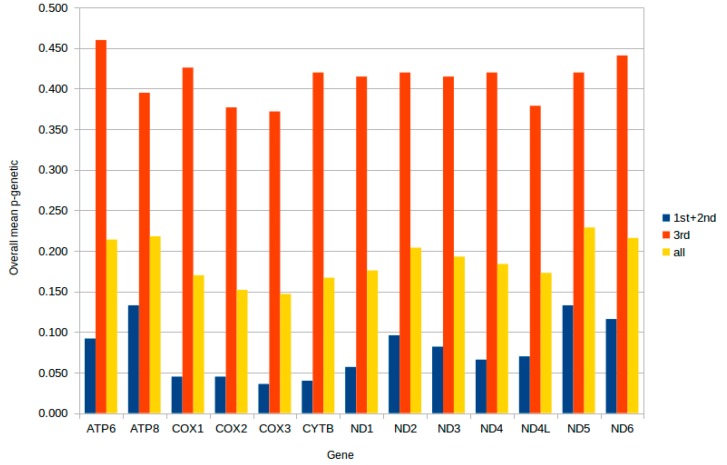
Overall mean p-genetic distance of 19 Sciaenidae species for each of 13 protein genes. They were calculated based on the first and second nucleotide positions, on the third nucleotide position of amino acid codons, and on the full sequence of the 19 Sciaenidae species, respectively. The 19 Sciaenidae species were *A. amoyensis* (KM257863.1), *N. coibor* (KM 233452.1), *P. diacanthus* (KM257722.1), *A. japonicus* (NC_017610.1), *Bahaba taipingensis* (NC_018347.1), *Chrysochir aureus* (NC_016987.1), *Collichthys lucidus* (NC_014350.1), *C. niveatus* (NC_014263.1), *Dendrophysa russelii* (NC_017606.1), *Johnius belangerii* (NC_022464.1), *J. grypotus* (NC_021130.1), *Larimichthys crocea* (NC_011710.1), *L. polyactis* (NC_013754.1), *Miichthys miiuy* (NC_014351.1), *N. albiflora* (NC_015205.1), *N. miichthioides* (NC_029875.1), *Pennahia argentata* (NC_015202.1), *P. macrocephalus* (NC_031409.1), and *Sciaenops ocellatus* (NC_016867.1).

We evaluated the conservation of mtDNA genes based on the overall p-genetic distance among 19 Sciaenidae species (Figure 4) [58]. Of the 13 PCGs, the *COXIII* gene has the lowest overall p-genetic distance (0.036) and the *ATP8* and *ND5* genes had the highest value (0.133) based on data of the first and second nucleotides of codons. A full-length sequence comparison of each gene resulted in the lowest value for *COXIII* (0.147) and the highest for *ND5* (0.229). These data were consistent with the values based on data of the first and second nucleotides of codons. According to these results, *ND5* most likely had the fastest evolutionary rate among Sciaenidae species, while *COXIII* had the lowest. For the third or wobble nucleotide, all genes had a high overall p-genetic distance value, ranging from 0.372 to 0.460. As was the case for the other fishes, most of the differences in the mtDNA PCGs occurred at the wobble position [48].

### 2.4. Mitochondrial Gene Codon Usage

**Figure 5:**
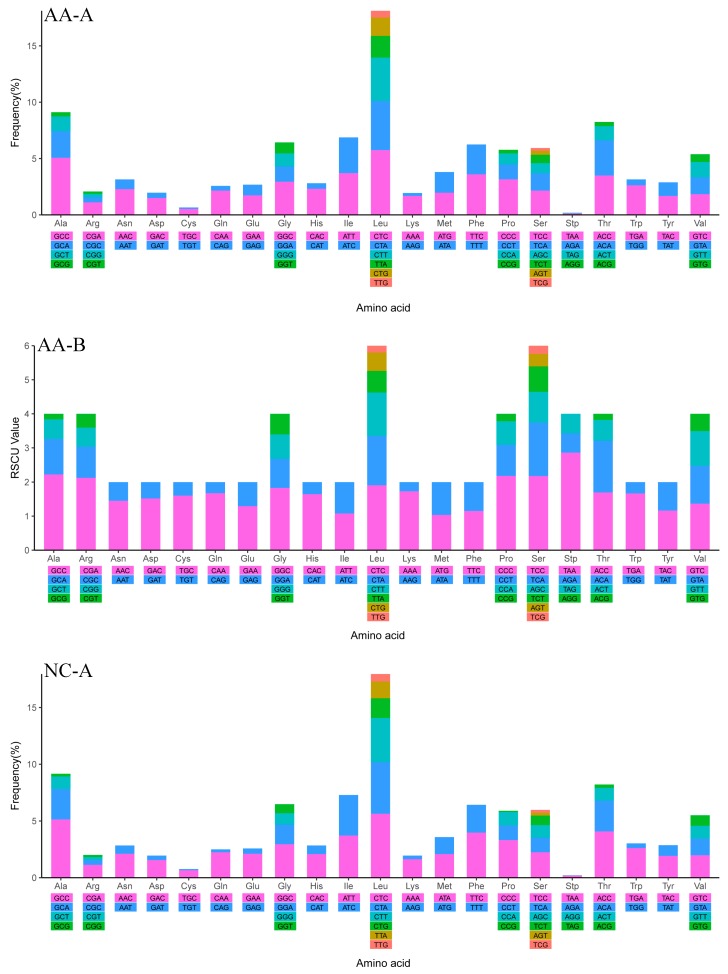

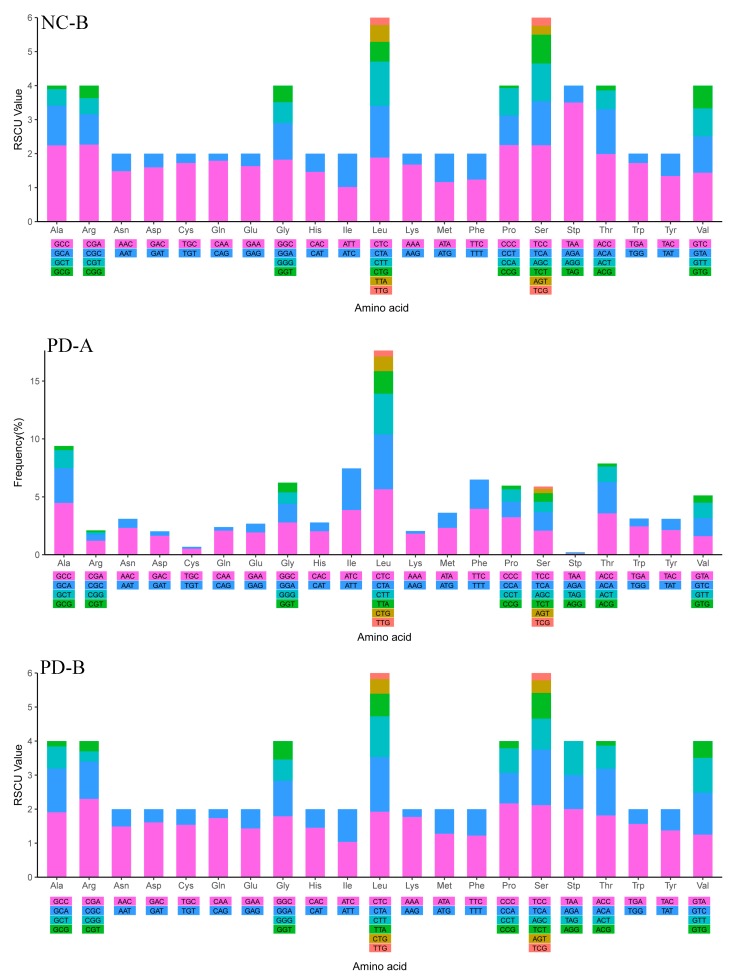
Codon Frequency (**A**) and RSCU (Relative Synonymous Codon Usage) (**B**) of mitochondrial genome for *Argyrosomus amoyensis* (AA), *Nibea coibor* (NC), and *Protonibea diacanthus* (PD).

The amino acids Leu and Ser were utilized by six different codons, while all other amino acids were encoded by either two or four. The frequencies in the PCGs of Leu (CTC, CTA) and Ala (GCC) were the most frequently used and Ser (TCG), Arg (CGT), and Cys (TGT) were least frequently used. However, relative synonymous codon usage (RSCU) analysis revealed that the codons coding Ala (GCC), Pro (CCC), Ser (TCC), and Arg (CGA) were the most frequently present, while those coding Pro (CCG), Leu (TTG), Thr (ACG), and Ala (GCG) were rare (Table S1 and Figure 5). Therefore, the codon frequency and RSCU were totally different indexes to value the mitochondrial gene codon usage. Only when the species had a similar codon usage preference would codon distribution and amino acid content be consistent among them. Moreover, differences in A+T content and the AT and GC skew of the PCGs were reflected in the codon usage (Table 1; Figure 2). They were closely related and mutually consistent [47,48,53]. The RSCU values indicated that codons with A or C in the third position were more used than T or G, so the codons NNA and NNC were in majority, while the synonymous codons NNT and NNG were in the minority.

### 2.5. Transfer and Ribosomal RNA Genes

The mitogenomes of the three croakers contained the 22 *tRNA* genes that are typically found in metazoan mitogenomes. They varied in size from 66 bp (*tRNA-Cys*) to 75 bp (*tRNA-Lys*) and the anticodons were identical to those reported in other vertebrates. Serine were determined by two types of anticodon (TGA, GCT) and leucine by TAG and TAA. With the exception of *tRNA-Ser* (GCT) of *N. coibor*, *tRNA-Ser* (GCT), and *tRNA-Asn* (GTT) of *P. diacanthus*, all of the 22 *tRNA* genes were predicted to be folded into canonical cloverleaf secondary structures, although there were numerous non-complementary and T-G base pairs in the stem regions. Stem mismatches seemed to be a common phenomenon for mitochondrial *tRNA* genes and are probably repaired via a post-transcriptional editing process [59]. The *tRNA-Ser* (GCT) found in the *N. coibor* and *P. diacanthus* mitogenomes had no recognizable DHU stem, which was the case for almost all vertebrate mitogenomes [60,61]. This *tRNA-Ser* has been previously described as a pseudogene to explain its unusual structure. That suggested that it could also perform the function by adjusting its structural conformation to fit the ribosome in a similar way to that of usual *tRNAs* in the ribosom [62].

As in all other mitogenomes described so far, two rRNA genes were present, a small (12S) and a large (16S) subunit. The *12S rRNA* gene ranged in size from 907 to 953 bp and the *16S rRNA* gene from 1696 to 1720 bp. Both genes were located on the H-strand between *tRNA-Phe* (GAA) and *tRNA-Leu* (TAA), separated by *tRNA-Val* as is the case in most vertebrates [54].

The AT content of the *12S rRNA* gene was between 51.42% and 51.95% with an AT-skew of 0.1846–0.2 and a GC-skew of −0.0842 to −0.0502. The AT content of the *16S rRNA* gene was between 52.41% to 53.26% with an AT-skew of 0.2174–0.2251 and a GC-skew of −0.1692 to −0.1339. The A and C content was more prevalent in the *12S rRNA* and *16S rRNA* genes of the three croakers, as has been reported in other bony fish [63]. That was similar to the entire mitogenome, and different with the PCGs. This implied that different kinds of mitogenome genes preferred to different bias.

### 2.6. CG View Comparison Tool (CCT) Map

**Figure 6:**
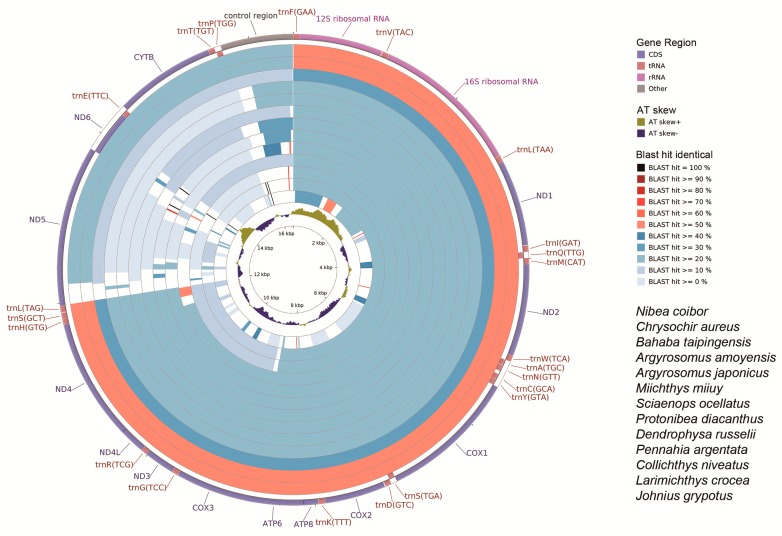
Graphical map of the BLAST results showing nucleotide identity between the complete mitochondrial genomes of *Nibea albiflora* (NC_015205) (Perciformes, Sciaenidae) and other Sciaenidae species, using the CGView comparison tool (CCT). CCT arranges BLAST result in an order where sequence that is most similar to the reference (*N. albiflora*) is placed closer to the outer edge of the map. Note: gene region, Blast identity, and AT skew are shown from outside to inside. The species from outside to inside as follows, respectively: *Nibea coibor*, *Chrysochir aureus*, *Bahaba taipingensis*, *Argyrosomus amoyensis*, *A. japonicas*, *Miichthys miiuy*, *Sciaenops ocellatus*, *Protonibea diacanthus*, *Dendrophysa russelii*, *Pennahia argentata*, *Collichthys niveatus*, *Larimichthys crocea*, and *Johnius grypotus*.

**Figure 7:**
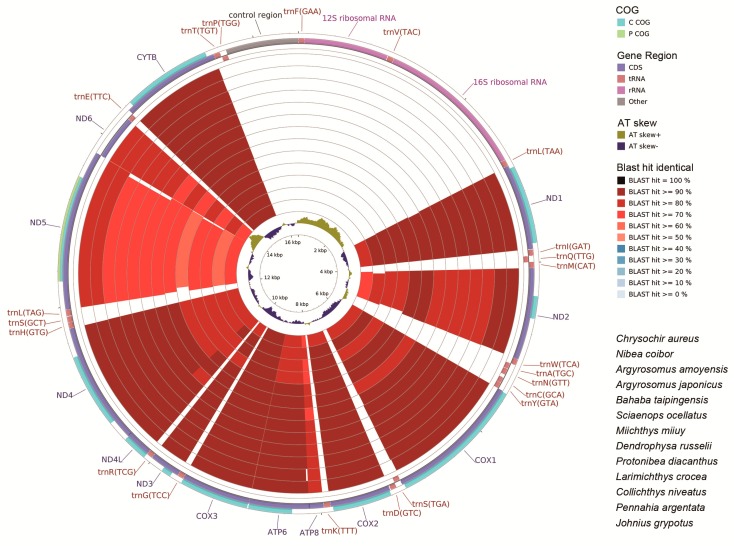
Graphical map of the BLAST results showing the mitochondrial cds (coding DNA sequence) identity between *Nibea albiflora* (NC_015205) (Perciformes, Sciaenidae) and other Sciaenidae species, using the CGView comparison tool (CCT). CCT arranges BLAST result in an order where sequence that is most similar to the reference (*N. albiflora*) is placed closer to the outer edge of the map. Note: COG (Clusters of Orthologous Groups of proteins), gene region, Blast identity, and AT skew are shown from outside to inside. The species from outside to inside as follows, respectively: *Chrysochir aureus*, *Nibea coibor*, *Argyrosomus amoyensis*, *A. japonícus*, *Bahaba taipingensis*, *Sciaenops ocellatus*, *Miichthys miiuy*, *Dendrophysa russelii*, *Protoníbea diacanthus*, *Larimichthys crocea*, *Collíchthys niveatus*, *Pennahia argentata*, and *Johnius grypotus*.

We compared the complete mitochondrial genomes of *N. albiflora* (Sciaenidae) with other available mitogenomes of the family Sciaenidae. Both the nucleotide (Figure 6) and coding DNA sequences (Figure 7) of the mitochondrial genomes illustrated the gene similarity between the three croakers sequenced in our study and the other Sciaenidae species, with the *N. albiflora* randomly selected as the reference sequence.

There was less identity in the mitogenome nucleotides than the coding DNA sequences (compare Figure 6 and Figure 7). In the 37 genes and the control region (D-loop) of the mitogenome, *ND5* was the least conservative in all species. This was appropriate for the phylogenetic and evolutional analyses within the family Sciaenidae. On the contrary, *Cytb* was the most conservative, which is appropriate for the analyses above the family Sciaenidae (Figure 7).

### 2.7. Phylogenetic Analyses

**Figure 8:**
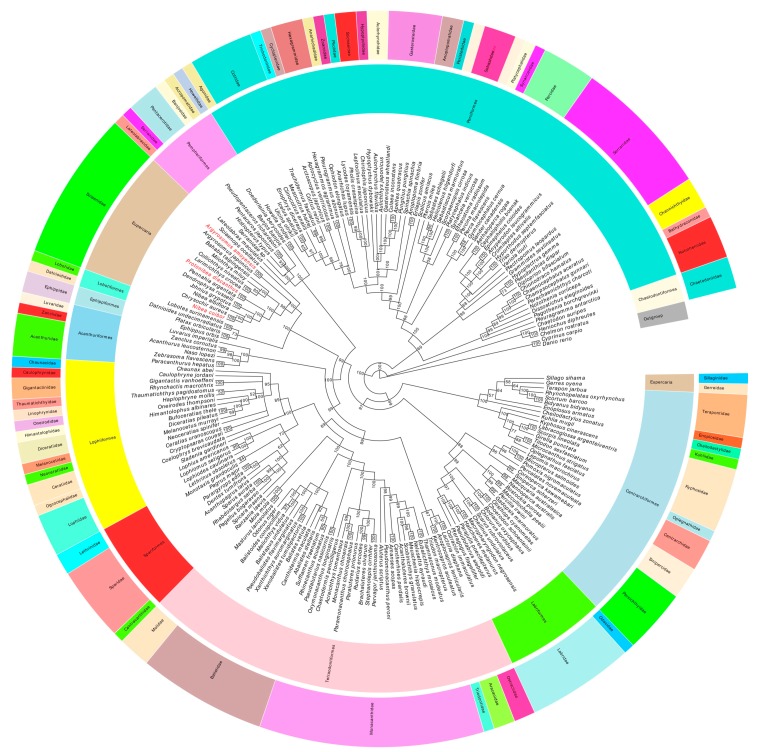
Bayesian inference (BI) tree inferred from the amino acid sequences of 13 PCGs of 204 species of Series Eupercaria including 2 out-groups (*Cyprinus carpio* and *Danio rerio*). The numbers at the nodes showed the Bayesian posterior probabilities. The species in red Latin name indicated the sequences generated in this study.

Based on the concatenated alignment of 13 PCGs amino acid sequences of 204 species in the Series Eupercaria analyzed in this study (Table S3), Bayesian and ML analyses produced almost identical topologies with similar branch lengths and strong bootstraps (ML analysis) and posterior probabilities (Bayesian inference) values (Figure 8). We preferred the phylogenetic analyses based on the 13 amino acid sequences of PCGs rather than the mitochondrial genome nucleotides because the 204 species of the Series Eupercaria belonged to different orders and possessed wide-ranging taxonomies. By multiple sequence alignment of their amino acid and nucleotide sequences, we figured out that nucleotide sequences were more conservative and suitable for the phylogenetic analyses of the intensive taxonomy [64,65,66]. To summarize, whether the 13 amino acid sequences or nucleotides of the 13 PCGs of mitochondrial genome is appropriate for the phylogenetics dependent upon the specific species adopted.

The phylogenetic tree analysis indicated that all the species of the Sciaenidae clustered on the same branch, and all the posterior probabilities were 1 except for *Miichthys* (0.8041) and *Argyrosomus* (0.9884). Furthermore, the intergeneric and interspecific taxonomic positions were explicit and clear.

Unexpectedly, the overall taxonomy of the whole Sciaenidae branch based on the mitochondrial genome classification was not consistent with the traditional Chinese fish taxonomy references [7,67]. The family Sciaenidae is traditionally classified as Perciformes consistent with Fishbase. However, the NCBI taxonomy software based on the DNA sequences revealed that the Sciaenidae position in the Eupercaria is uncertain. Algorithms used in the DeepFin program based on 20 nuclear genes and a mitochondrial gene show that the Sciaenidae have an uncertain placement in the Percomorpharia [32,36,68]. In addition, the Percomorpharia are at a higher taxonomic level than the Eupercaria. Therefore, although there are great differences between traditional DNA molecular marker taxonomies, the analyses using the coding gene sequences from the mitochondrial genome in our manuscript were closer to the ones in NCBI and DeepFin.

The Sciaenidae have evolved phyletically and were closer to the orders Lobotiformes and Ephippiformes and at the same classification level (Figure 8). Tracing back at the branch nodes of the evolutionary tree from Sciaenidae to Perciformes, they have experienced six branch nodes with node bootstrap values of 1, 1, 0.9982, 0.9791, 0.9974, and 1. Sciaenidae are the most distant from the order Perciformes and the high posterior probabilities of each branch support that the Sciaenidae are an independent branch, isolated from the order Perciformes. Sciaenidae have been included in the orders Acanthuriformes, Acanthuridae, Emmelichthyidae, Luvaridae, and Zanclidae [31]. However, there are exceptions to this scheme where the placement of Emmelichthyidae and Sciaenidae in the Acanthuriformes was not supported, consistent with our viewpoint [32].

In summary, synthetically considering the taxonomy of Sciaenidae and the relationship between the Sciaenidae and the Perciformes, we predict that the Sciaenidae belongs to a new unknown order rather than the extant order Perciformes.

### 2.8. Divergence Times of the Series Eupercaria

**Figure 9:**
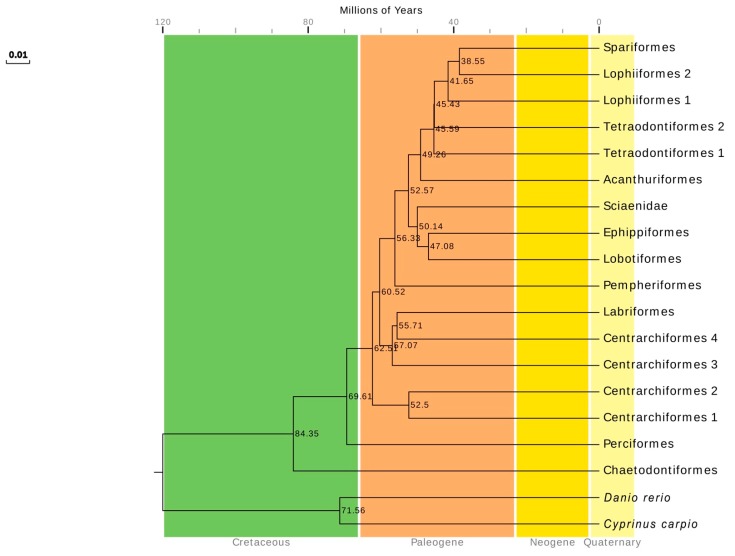
Chronogram for the 204 species of Series Eupercaria including 2 out-groups (*Cyprinus carpio* and *Danio rerio*) based on the Bayesian topology resulting from analysis of the 13 PCGs genes. Divergence times were estimated using three calibrations. Numbers near the nodes indicated the average divergence time estimated (million years, Mya). The geological time scales were Cretaceous, Paleogene, Neogene, and Quaternary, respectively.

We estimated divergence times based on phylogenetic trees constructed with the Bayesian method, and the species differentiation time estimated using the ML method of RelTime MEGA 7.0 [69] (Figure 9). In the ProtTest 3 model preferences [70], the model MtMam+I+G+F was optimal with an AIC value of 650,366.12. The model JTT+I+G+F was suboptimal with an AIC value of 653,040.01, similar to the one for model MtMam+I+G+F. Our software did not support the MtMam+I+G+F model so we used the JTT+I+G+F model for the molecular clock.

The divergence time calculations indicated that the Perciformes began to differentiate from other species about 73.49 million years ago and then subsequently evolved as an independent branch. The Sciaenidae differentiation from the Ephippiformes and Lobotiformes lagged far behind Perciformes and began around 51.52 million years ago. Therefore, there was a large gap of 21.97 million years in the differentiation initiation times of the Perciformes and Sciaenidae. The Sciaenidae initiated their differentiation after 4 important differentiation nodes at 65.76, 63.38, 60.15, and 55.92 million years ago and all lagged behind Perciformes differentiation. In summary, the Sciaenidae was an independent branch of fish evolution that was entirely independent with, and lagged far behind, Perciformes differentiation.

## 3. Materials and Methods

### 3.1. Sampling and Materials

The wild specimens of the three croakers (*N. coibor*, *P. diacanthus*, and *A. amoyensis*) were collected from the South China Sea near Guangdong province (N23°19′, E117°09′), China, on 3–18 July 2014 (identification code: NC_005072014, PD_006072014, AA_007072014). The dorsal muscle was preserved in 95% ethanol and stored at −70 °C until they were used for DNA extraction. Voucher specimens were deposited in the Germplasm Resources Lab, College of Marine Sciences, South China Agricultural University, Guangzhou, China (accession numbers: 005072014, 006072014, 007072014). Genomic DNA was isolated from muscle tissue as previously described [71]. Total DNA was eluted in sterile deionized water and was stored at −20 °C. All animal experiments were conducted in accordance with the guidelines and approval of the Animal Research and Ethics Committees of South China Agricultural University.

### 3.2. Library Preparation for Sequencing

DNA sample quality and quantity were characterized by gel electrophoresis and the Nano-Drop 2000 spectrometer (Thermo Scientific, Waltham, MA, USA). The high-quality genomic DNA were used to prepare DNA library, with insert sizes of 500 bp for paired-end sequencing. Paired-end reads of 100 bp were generated on an Illumina HiSeq2500 (Illumina, Inc., San Diego, CA, USA) using sequencing protocols provided by the manufacturer.

### 3.3. Sequence Analysis and Annotation

Illumina paired-end sequencing reads were filtered on quality values, and the low quality bases (quality < 20, perror > 0.01) of 5′ upstream and 3′ downstream were trimmed. The mitochondrial genome sequencing reads were captured by Sciaenidae mitochondrial genomes on NCBI with bowtie2 (Http://Bowtie-Bio.Sourceforge.Net/Bowtie2/Index.Shtml) aligner. De novo assembly with paired-end sequencing reads was determined using SOAPdenovo2 (http://soap.genomics.org.cn/soapdenovo.html). The protein-coding regions and ribosomal genes were identified using the Basic Local Alignment Search Tool (https://blast.ncbi.nlm.nih.gov/Blast.cgi) [72]. Transfer RNA genes were annotated using *tRNA* scan-SE v.2.0 (http://lowelab.ucsc.edu/tRNAscan-SE/) using a Cove score cutoff of 0.1 coupled with ARWEN software (http://mbio-serv2.mbioekol.lu.se/ARWEN/) [73,74] and then confirmed using MitoFish (http://mitofish.aori.u-tokyo.ac.jp/) to confirm the annotation. Species maps were drawn using OGDRAW (http://ogdraw.mpimp-golm.mpg.de/).

To describe base composition, strand asymmetry was calculated using the following formulas: AT skew = [A − T]/[A + T] and GC skew = [G − C]/[G + C] [75]. Repeat sequences were identified found using Spectral Repeat Finder v1.1 [76]. Long repeat analysis used the web-based REPuter (http://bibiserv.techfak.uni-bielefeld.de/reputer/) and included forward, reverse, and tandem repeats with minimal lengths of 30 bp and edit distances of <3 bp [77].

Codon usage was determined for all PCGs. The Relative Synonymous Codon Usage (RSCU) was obtained using MEGA 7 software [69]. Statistical analyses of the distributions and visualization of codon usage in the form of heat maps were conducted using R language with RSCU values, a measure of non-uniform usage of synonymous codons in a coding sequence [78]. The RSCU value was the number of times a particular codon was observed relative to the number of times that the codon would be observed for a uniform synonymous codon usage (i.e., all codons for a given amino acid exhibiting similar probabilities). The RSCU value in the absence of any codon usage bias is 1.00. A codon used less frequently than expected will have RSCU values <1.00, whereas codons used more frequently than expected will have RSCU values >1.00.

### 3.4. Phylogenetic Analyses

We conducted phylogenetic analyses based on 204 mitochondrial genomes sequences of the Series Eupercaria (Table S3). *Cyprinus carpio* and *Danio rerio* (Cypriniformes, Cyprinidae) were used as out-groups. All sequences were deposited in GenBank. We aligned amino acid sequences of PCGs in the 204 species using the MUSCLE program in MEGA 7. Our alignments of individual genes excluded the stop codon and the third codon.

Phylogenetic analysis was performed using the Maximum Likelihood (ML) and Bayesian Inference (BI) methods. The best model MtMam+I+G+F based on the amino acid sequences in this study was selected using AIC (Akaike Information Criteria). Minimum and best values were fitted with ProtTest 3 with optimized parameters [70]. ML analysis was conducted using the RAxML 8.1.5 with 1000 bootstrap replicates based on the best-scoring protein substitution model determined automatically by the software [79]. Bayesian phylogenetic analysis with 10,000,000 generations was carried out with MrBayes 3.2.6 software [80]. Four independent Markov chains were used at the same time with sampling every 100 generations. The BI Tree was reliable since the standard deviation of split frequencies was below 0.01. The phylogenetic trees were drawn with the Evolview (http://www.evolgenius.info/evolview/) [81,82]. Fishbase (http://fishbase.org), the NCBI taxonomy server (http://www.ncbi.nlm.nih.gov/taxonomy/), and DeepFin (http://deepfin.org/) were used in the additional analyses.

The divergence times of the Series Eupercaria was estimated using MEGA 7.0 [83] with the RelTime-ML method and JTT+F+I+G modeling. Divergence times were presented as in the Time Tree database (http://www.timetree.org/) [69]. Bayesian phylogenetic data were input to MEGA 7.0 to generate divergence times that ensured consistency between the phylogenetic tree and divergence times. 

Sequence differences between the three croakers and those from other Sciaenidae species using the *Nibea albiflora* (NC_015205) as the reference sequence. Sequences were aligned using BLAST and annular genetic similarity mapping was visualized using the CGView Comparison Tool (http://stothard.afns.ualberta.ca/downloads/CCT).

## 4. Conclusions

We have here presented the characterization of the complete mitochondrial genome sequences of three croakers in the order Perciformes, family Sciaenidae. Gene arrangement and distribution of the three croakers are canonically identical and consistent with other vertebrates. The Sciaenidae is an independent branch because it is isolated from the order Perciformes and does not belong to any currently known order. The differentiation of the independent Sciaenidae is lagging far behind that of Perciformes. This study presents a novel insight into the phylogenetic analysis of the family Sciaenidae from the order Perciformes and facilitates additional studies on the evolution and phylogeny of the Series Eupercaria.

## Figures and Tables

**Table 1:** Summary of the base composition of the mitogenomes at each codon position of the concatenated 13 protein-coding genes (PCGs) across 19 species of family Sciaenidae.

Species	Accession Number	Length (bp)	Entire Genome						Protein-Coding Gene					
			A (%)	T (%)	C (%)	G (%)	AT (%)	AT-Skew	GC-Skew	Length (aa)	AT (%) (all)	AT (%) (3rd)	AT-Skew	GC-Skew
*Protonibea diacanthus*	KM257722.1	16,535	27.54	25.34	31.09	16.03	52.88	0.0416	−0.3195	3795	51.83	47.28	−0.0123	−0.3904
*Argyrosomus amoyensis*	KM257863.1	16,490	26.96	25.31	31.02	16.71	52.27	0.0316	−0.2998	3802	51.23	51.43	−0.0205	−0.371
*Nibea coibor*	KM233452.1	16,502	26.88	25.19	31.62	16.31	52.07	0.0324	−0.3193	3791	50.91	49.3	−0.0191	−0.3955
*Johnius belangerii*	NC_022464.1	19,154	24.96	36.79	17.59	20.67	61.75	−0.1916	0.0805	3802	60.89	62.19	−0.3011	0.0273
*Pennahia argentata*	NC_015202.1	16,485	27.46	26.33	30.18	16.04	53.79	0.021	−0.3059	3802	52.98	54.92	−0.0357	−0.3747
*Argyrosomus japonicus*	NC_017610.1	16,496	26.99	25.29	31.02	16.69	52.28	0.0326	−0.3002	3802	51.24	51.43	−0.0196	−0.372
*Dendrophysa russelii*	NC_017606.1	16,626	27.28	26.12	30.4	16.2	53.4	0.0217	−0.3048	3801	52.48	54.02	−0.0302	−0.3797
*Pennahia macrocephalus*	NC_031409.1	16,508	27.51	25.28	31.22	15.99	52.79	0.0423	−0.3227	3793	51.7	47.92	−0.009	−0.3975
*Bahaba taipingensis*	NC_018347.1	16,500	27.55	25.15	31.41	15.9	52.7	0.0457	−0.3279	3802	51.67	51.06	0	−0.4051
*Nibea albiflora*	NC_015205.1	16,499	26.4	25.88	30.81	16.91	52.28	0.0099	−0.2913	3797	51.26	49.61	−0.0477	−0.3604
*Larimichthys crocea*	NC_011710.1	16,466	27.55	25.46	30.69	16.3	53.01	0.0393	−0.3062	3800	52.2	52.26	−0.0169	−0.3745
*Collichthys lucidus*	NC_014350.1	16,442	27.95	25.63	30.69	15.73	53.58	0.0434	−0.3223	3801	52.98	53.49	−0.0081	−0.3909
*Sciaenops ocellatus*	NC_016867.1	16,500	27.47	25.47	30.72	16.35	52.94	0.0378	−0.3054	3803	52	51.42	−0.0118	−0.3803
*Nibea miichthioides*	NC_029875.1	16,490	26.96	25.32	31.04	16.69	52.28	0.0313	−0.3006	3802	51.25	51.54	−0.0189	−0.3728
*Johnius grypotus*	NC_021130.1	18,523	24.99	36.18	18.57	20.27	61.17	−0.183	0.0439	3803	60.61	62.51	−0.2738	0.0007
*Collichthys niveatus*	NC_014263.1	16,469	27.59	24.96	31.3	16.16	52.55	0.0499	−0.319	3801	51.61	51.76	0.0003	−0.3876
*Larimichthys polyactis*	NC_013754.1	16,470	27.55	25	31.27	16.18	52.55	0.0486	−0.318	3801	51.65	51.84	−0.0012	−0.3866
*Chrysochir aureus*	NC_016987.1	16,505	26.93	25.17	31.64	16.25	52.1	0.0337	−0.3214	3802	50.9	49.3	−0.0199	−0.3947
*Miichthys miiuy*	NC_014351.1	16,493	27.49	24.43	32.19	15.89	51.92	0.059	−0.339	3802	50.63	49.93	0.0124	−0.4165

**Table 2:** Summary of gene/element feature of *A. amoyensis*, *N. coibor*, and *P. diacanthus*.

Gene/Element	Strand	Size (bp)	GC_Percent (%)	Amino Acids (aa)	Inferred Initiation Codon	Inferred Termination Codon	One Letter Code	Anti-Codon	Intergenic Nucleotide * (bp)
*tRNA-Phe*	H	68–70	40.58–45.50				F	GAA	0–45
*12S* *rRNA*	H	907–953	48.05–48.58						0
*tRNA-Val*	H	71–72	46.48–50.00				V	TAC	0
*16S* *rRNA*	H	1696–1720	46.74–47.59						0
*tRNA-Leu*	H	74	44.59–48.65				L	TAA	0
*ND1*	H	975	48.00–49.13	324	ATG, GTG	TAG, TAA			4
*tRNA-Ile*	H	70	42.86–51.43				I	GAT	−1
*tRNA-Gln*	L	71	43.66–46.48				Q	TTG	−1
*tRNA-Met*	H	69	39.13–44.93				M	CAT	0
*ND2*	H	1045–1046	47.90–50.72	348	ATG	TA, T			0
*tRNA-Trp*	H	71	49.30–50.70				W	TCA	0–1
*tRNA-Ala*	L	69	37.68–42.03				A	TGC	2
*tRNA-Asn*	L	72–73	45.21–50.68				N	GTT	34–36
*tRNA-Cys*	L	66	43.94–53.03				C	GCA	0
*tRNA-Tyr*	L	70	45.71–50.00				Y	GTA	1
*COXI*	H	1557	49.13–49.90	518	ATG	AGA			−5
*tRNA-Ser*	L	71	46.48–50.70				S	TGA	3
*tRNA-Asp*	H	69	40.58–46.38				D	GTC	8
*COXII*	H	687–691	44.69–45.59	228–230	ATG	T, AGA			0–5
*tRNA-Lys*	H	74–75	46.67–50.00				K	TTT	1
*ATP8*	H	168	40.48–46.43	55	ATG	TAA			(−10)–17
*ATP6*	H	657–684	47.79–49.12	218–227	ATG, ATA, GTG	TA, TAA			0–1
*COXIII*	H	785–786	48.03–50.25	261	ATG	TA, TAA			(−1)–0
*tRNA-Gly*	H	71	35.21–36.62				G	TCC	0
*ND3*	H	349	50.14–52.44	116	ATG	T			0
*tRNA-Arg*	H	69	36.23–37.68				R	TCG	0
*ND4L*	H	297	49.83–53.20	98	ATG	TAA			−7
*ND4*	H	1381	48.08–49.67	460	ATG	T			0
*tRNA-His*	H	69–72	34.78–36.11				H	GTG	(−1)–0
*tRNA-Ser*	H	68–71	54.41–57.35				S	GCT	2–5
*tRNA-Leu*	H	73	43.84–49.32				L	TAG	0–15
*ND5*	H	1824–1839	47.47–47.80	607–612	ATG, ATC	TAA, TAG			−4
*ND6*	L	522	45.40–50.77	173	ATG	TAA, TAG			0–1
*tRNA-Glu*	L	69	39.13–46.38				E	TTC	3–5
*Cytb*	H	1137–1141	48.72–50.57	378–380	ATG	T, TAA			0
*tRNA-Thr*	H	72	52.78–56.94				T	TGT	3–5
*tRNA-Pro*	L	70	34.29–38.57				P	TGG	0
D-loop	H	821–838	35.92–37.50						0

* As for the adjacent genes, positive number represented spaced, negative number indicated overlap and zero indicated continuous.

## Supplementary Materials

Supplementary materials can be found at http://www.mdpi.com/1422-0067/19/6/1741/s1.

## References

[1] Lal Mohan R.S. (1984). Fao Species Identification Sheets for Fishery Purposes.

[2] Nguyen H.P., Trong P.L., Nguyen N.T., Nguyen P.D., Do T.N.N., Nguyen V.L. (1995). Checklist of Marine Fishes in Vietnam.

[3] Sasaki K. (2001). Fao Species Identification Guide for Fishery Purposes.

[4] Shao Y.Q., Zhang D.C., Su T.F., Lv J.L., Jiang S.G. (2006). The construction of pituitary gland cDNA library of nibea coibor. South China Fish. Sci..

[5] Jian L.J., Yang Y.K., Liu X.D., Chen Q.K., Wang Z.Y. (2013). The cross breeding and genetic analysis of hybrids of *Larimichthys crocea* (♀) and *Nibea miichthioides* (♂). J. Fish. China.

[6] Meng Q.W. (1996). Fish Taxonomy.

[7] Cheng Q.T., Zheng B.S. (1987). Systematic Synopsis of Chinese Fishes.

[8] Curole J.P., Kocher T.D. (1999). Mitogenomics: Digging deeper with complete mitochondrial genomes. Trends Ecol. Evol..

[9] Podsiadlowski L., Kohlhagen H., Koch M. (2007). The complete mitochondrial genome of *Scutigerella causeyae* (myriapoda: Symphyla) and the phylogenetic position of symphyla. Mol. Phylogenet. Evol..

[10] Boore J.L. (1999). Animal mitochondrial genomes. Nucleic Acids Res..

[11] Kartavtsev Y.P., Jung S.O., Lee Y.M., Byeon H.K., Lee J.S. (2007). Complete mitochondrial genome of the bullhead torrent catfish, *Liobagrus obesus* (siluriformes, amblycipididae): Genome description and phylogenetic considerations inferred from the CYT b and 16s rRNA genes. Gene.

[12] Wolstenholme D.R. (1992). Animal mitochondrial DNA: Structure and evolution. Int. Rev. Cytol..

[13] Moritz C., Dowling T., Brown W. (1987). Evolution of animal mitochondrial DNA: Relevance for population biology and systematics. Annu. Rev. Ecol. Syst..

[14] Stepien C.A., Kocher T.D. (1997). Molecules and Morphology in Studies of Fish Evolution. Molecular Systematics of Fishes.

[15] Miya M., Nishida M. (2000). Use of mitogenomic information in teleostean molecular phylogenetics: A tree-based exploration under the maximumparsimony optimality criterion. Mol. Phylogenet. Evol..

[16] Jakhar J.K., Basu S., Sasidharan S., Chouksey M.K., Gudipati V. (2014). Optimization of process parameters for gelatin extraction from the skin of blackspotted croaker using response surface methodology. J. Food Sci. Technol..

[17] Lakra W.S., Goswami M., Gopalakrishnan A. (2009). Molecular identification and phylogenetic relationships of seven Indian *Sciaenids* (pisces: Perciformes, sciaenidae) based on 16s rRNA and cytochrome c oxidase subunit i mitochondrial genes. Mol. Biol. Rep..

[18] Liu L., Yang H., Yang Z., Zhao H., Sun J., Xiao S., Yang X., Li G. (2016). The complete mitochondrial genome of the blackspotted croaker *Protonibea diacanthus* (perciformes, sciaenidae). Mitochondrial DNA. Part A.

[19] Moravec F., Barton D.P. (2015). Two gonad-infecting species of *Philometra* (nematoda: Philometridae) from marine fishes off the northern coast of Australia. Parasite.

[20] Shan B., Zhao L., Gao T., Lu H., Yan Y. (2016). The complete mitochondrial genome of *Nibea coibor* (perciformes: Sciaenidae). Mitochondrial DNA. Part A.

[21] Taillebois L., Crook D., Saunders T., Ovenden J. (2016). The complete mitochondrial genome of the black jewfish *Protonibea diacanthus* (perciformes: Sciaenidae). Mitochondrial DNA. Part A.

[22] Yang H., Zhao H., Sun J., Yang Z., Xiao S., Li G., Liu L. (2016). The complete mitochondrial genome of the amoy croaker *Argyrosomus amoyensis* (Perciformes, Sciaenidae). Mitochondrial DNA Part A.

[23] Zhang D., Shao Y., Jiang S., Li J., Xu X. (2009). *Nibea coibor* growth hormone gene: Its phylogenetic significance, microsatellite variation and expression analysis. Gen. Comp. Endocrinol..

[24] Cheng Y., Xu T., Jin X., Shi G., Wang R. (2012). The complete mitochondrial genome of silver croaker *Argyrosomus argentatus* (perciforems; sciaenidae): Genome characterization and phylogenetic consideration. Molekuliarnaia Biologiia.

[25] Xu T., Tang D., Jin X. (2015). A surprising arrangement pattern and phylogenetic consideration: The complete mitochondrial genome of belanger’s croaker *Johnius belangerii* (percoidei: Sciaenidae). Mitochondrial DNA.

[26] Barbosa A.J., Sampaio I., Schneider H., Santos S. (2014). Molecular phylogeny of weakfish species of the *Stellifer* group (sciaenidae, perciformes) of the western south Atlantic based on mitochondrial and nuclear data. PLoS ONE.

[27] Santos S., Gomes Mde F., Ferreira A.R., Sampaio I., Schneider H. (2013). Molecular phylogeny of the western south Atlantic sciaenidae based on mitochondrial and nuclear data. Mol. Phylogenet. Evol..

[28] Cheng Y., Wang R., Sun Y., Xu T. (2012). The complete mitochondrial genome of the small yellow croaker and partitioned bayesian analysis of sciaenidae fish phylogeny. Genet. Mol. Biol..

[29] Cheng J., Ma G.Q., Song N., Gao T.X. (2012). Complete mitochondrial genome sequence of bighead croaker *Collichthys niveatus* (perciformes, sciaenidae): A mitogenomic perspective on the phylogenetic relationships of pseudosciaeniae. Gene.

[30] Cheng Y., Wang R., Xu T. (2011). The mitochondrial genome of the spinyhead croaker collichthys lucida: Genome organization and phylogenetic consideration. Mar. Genom..

[31] Nelson J.S., Grande T., Wilson M.V.H. (2016). Fishes of the World.

[32] Betancur R.R., Wiley E.O., Arratia G., Acero A., Bailly N., Miya M., Lecointre G., Orti G. (2017). Phylogenetic classification of bony fishes. BMC Evol. Biol..

[33] Miya M., Takeshima H., Endo H., Ishiguro N.B., Inoue J.G., Mukai T., Satoh T.P., Yamaguchi M., Kawaguchi A., Mabuchi K. (2003). Major patterns of higher teleostean phylogenies: A new perspective based on 100 complete mitochondrial DNA sequences. Mol. Phylogenet. Evol..

[34] Near T.J., Dornburg A., Eytan R.I., Keck B.P., Smith W.L., Kuhn K.L., Moore J.A., Price S.A., Burbrink F.T., Friedman M. (2013). Phylogeny and tempo of diversification in the superradiation of spiny-rayed fishes. Proc. Natl. Acad. Sci. USA.

[35] Smith W.L., Wheeler W.C. (2006). Venom evolution widespread in fishes: A phylogenetic road map for the bioprospecting of piscine venoms. J. Hered..

[36] Betancur R.R., Broughton R.E., Wiley E.O., Carpenter K., Lopez J.A., Li C., Holcroft N.I., Arcila D., Sanciangco M., Cureton Ii J.C. (2013). The tree of life and a new classification of bony fishes. PLoS Curr..

[37] Liu L., Yang H., Zhao H., Sun J., Han X., Zhu S., Li G. (2016). The complete mitochondrial genome of the *Plectorhinchus cinctus* (teleostei, haemulidae). Mitochondrial DNA Part A.

[38] Yang H., Sun J., Zhao H., Chen Y., Yang Z., Li G., Liu L. (2016). The complete mitochondrial genome of the *Clarias fuscus* (Siluriformes, Clariidae). Mitochondrial DNA A.

[39] Yang H., Xie Z., Li S., Wu X., Peng C., Zhang Y., Lin H. (2016). The complete mitochondrial genome of the orange-spotted grouper *Epinephelus coioides* (perciformes, serranidae). Mitochondrial DNA Part A.

[40] Yang H., Zhang J.E., Luo H., Luo M., Guo J., Deng Z., Zhao B. (2016). The complete mitochondrial genome of the mudsnail *Cipangopaludina cathayensis* (gastropoda: Viviparidae). Mitochondrial DNA Part A.

[41] Yang H., Zhao H., Sun J., Chen Y., Liu L., Zhang Y., Liu L. (2016). The complete mitochondrial genome of the *Hemibarbus medius* (cypriniformes, Cyprinidae). Mitochondrial DNA Part A.

[42] Yang H., Zhao H., Sun J., Xie Z., Yang Z., Liu L. (2016). The complete mitochondrial genome of the *Culter recurviceps* (teleostei, cyprinidae). Mitochondrial DNA Part A.

[43] Yang H., Zhao H., Sun J., Zhang Y., Yang Z., Liu L. (2016). The complete mitochondrial genome of the *Hemibagrus wyckioides* (siluriformes, Bagridae). Mitochondrial DNA Part A.

[44] Yang H., Zhao H., Xie Z., Sun J., Yang Z., Liu L. (2016). The complete mitochondrial genome of the *Hemibagrus guttatus* (teleostei, Bagridae). Mitochondrial DNA Part A.

[45] Zhao H., Yang H., Sun J., Chen Y., Liu L., Li G., Liu L. (2016). The complete mitochondrial genome of the *Anabas testudineus* (perciformes, Anabantidae). Mitochondrial DNA Part A.

[46] Yang H., Zhang J.E., Guo J., Deng Z., Luo H., Luo M., Zhao B. (2016). The complete mitochondrial genome of the giant African snail *Achatina fulica* (mollusca: Achatinidae). Mitochondrial DNA Part A.

[47] Shi X., Tian P., Lin R., Huang D., Wang J. (2016). Characterization of the complete mitochondrial genome sequence of the globose head whiptail *Cetonurus globiceps* (gadiformes: Macrouridae) and its phylogenetic analysis. PLoS ONE.

[48] Zhuang X., Qu M., Zhang X., Ding S. (2013). A comprehensive description and evolutionary analysis of 22 grouper (perciformes, Epinephelidae) mitochondrial genomes with emphasis on two novel genome organizations. PLoS ONE.

[49] Francino M.P., Ochman H. (1997). Strand asymmetries in DNA evolution. Trends Genet. TIG.

[50] Perna N.T., Kocher T.D. (1995). Patterns of nucleotide composition at fourfold degenerate sites of animal mitochondrial genomes. J. Mol. Evol..

[51] Wu Q.L., Li Q., Gu Y., Shi B.C., van Achterberg C., Wei S.J., Chen X.X. (2014). The complete mitochondrial genome of *Taeniogonalos taihorina* (bischoff) (hymenoptera: Trigonalyidae) reveals a novel gene rearrangement pattern in the hymenoptera. Gene.

[52] Zhang H.L., Zeng H.H., Huang Y., Zheng Z.M. (2013). The complete mitochondrial genomes of three grasshoppers, *Asiotmethis zacharjini, Filchnerella helanshanensis* and *Pseudotmethis rubimarginis* (orthoptera: Pamphagidae). Gene.

[53] Chao Q.J., Li Y.D., Geng X.X., Zhang L., Dai X., Zhang X., Li J., Zhang H.J. (2014). Complete mitochondrial genome sequence of *Marmota himalayana* (rodentia: Sciuridae) and phylogenetic analysis within rodentia. Genet. Mol. Res. GMR.

[54] Satoh T.P., Miya M., Endo H., Nishida M. (2006). Round and pointed-head grenadier fishes (actinopterygii: Gadiformes) represent a single sister group: Evidence from the complete mitochondrial genome sequences. Mol. Phylogenet. Evol..

[55] Catanese G., Manchado M., Infante C. (2010). Evolutionary relatedness of mackerels of the genus scomber based on complete mitochondrial genomes: Strong support to the recognition of Atlantic scomber colias and pacific scomber japonicus as distinct species. Gene.

[56] Lynch M., Koskella B., Schaack S. (2006). Mutation pressure and the evolution of organelle genomic architecture. Science.

[57] Yang Z., Bielawski J.P. (2000). Statistical methods for detecting molecular adaptation. Trends Ecol. Evol..

[58] Xia J., Xia K., Jiang S. (2008). Complete mitochondrial DNA sequence of the yellowfin seabream acanthopagrus latus and a genomic comparison among closely related sparid species. Mitochondrial DNA.

[59] Lavrov D.V., Brown W.M., Boore J.L. (2000). A novel type of RNA editing occurs in the mitochondrial trnas of the centipede *Lithobius forficatus*. Proc. Natl. Acad. Sci. USA.

[60] Cui P., Ji R., Ding F., Qi D., Gao H., Meng H., Yu J., Hu S., Zhang H. (2007). A complete mitochondrial genome sequence of the wild two-humped camel (camelus bactrianus ferus): An evolutionary history of camelidae. BMC Genom..

[61] Zhou Y., Zhang J.Y., Zheng R.Q., Yu B.G., Yang G. (2009). Complete nucleotide sequence and gene organization of the mitochondrial genome of *Paa spinosa* (anura: Ranoidae). Gene.

[62] Ohtsuki T., Kawai G., Watanabe K. (2002). The minimal trna: Unique structure of ascaris suum mitochondrial tRNA(SER)(UCU) having a short t arm and lacking the entire d arm. FEBS Lett..

[63] Zardoya R., Meyer A. (1997). The complete DNA sequence of the mitochondrial genome of a “living fossil,” the coelacanth (latimeria chalumnae). Genetics.

[64] Li T., Yang J., Li Y., Cui Y., Xie Q., Bu W., Hillis D.M. (2016). A mitochondrial genome of *Rhyparochromidae* (hemiptera: Heteroptera) and a comparative analysis of related mitochondrial genomes. Sci. Rep..

[65] Li Y., Hu X.D., Yang R.H., Hsiang T., Wang K., Liang D.Q., Liang F., Cao D.M., Zhou F., Wen G. (2015). Complete mitochondrial genome of the medicinal fungus *Ophiocordyceps sinensis*. Sci. Rep..

[66] Liu G.H., Nadler S.A., Liu S.S., Podolska M., D’Amelio S., Shao R., Gasser R.B., Zhu X.Q. (2016). Mitochondrial phylogenomics yields strongly supported hypotheses for *Ascaridomorph nematode*s. Sci. Rep..

[67] Wu H.L., Shao G.Z., Lai C.F. (1999). Latin-Chinese International Fishes Manual.

[68] Betancur R.R., Orti G., Pyron R.A. (2015). Fossil-based comparative analyses reveal ancient marine ancestry erased by extinction in ray-finned fishes. Ecol. Lett..

[69] Kumar S., Stecher G., Tamura K. (2016). Mega7: Molecular evolutionary genetics analysis version 7.0 for bigger datasets. Mol. Biol. Evol..

[70] Darriba D., Taboada G.L., Doallo R., Posada D. (2011). Prottest 3: Fast selection of best-fit models of protein evolution. Bioinformatics.

[71] Green M.R., Sambrook J. (2012). Molecular Cloning: A Laboratory Manual.

[72] Iwasaki W., Fukunaga T., Isagozawa R., Yamada K., Maeda Y., Satoh T.P., Sado T., Mabuchi K., Takeshima H., Miya M. (2013). Mitofish and mitoannotator: A mitochondrial genome database of fish with an accurate and automatic annotation pipeline. Mol. Biol. Evol..

[73] Lowe T.M., Chan P.P. (2016). Trnascan-se on-line: Integrating search and context for analysis of transfer rna genes. Nucleic Acids Res..

[74] Laslett D., Canback B. (2008). Arwen: A program to detect tRNA genes in metazoan mitochondrial nucleotide sequences. Bioinformatics.

[75] Junqueira A.C., Lessinger A.C., Torres T.T., da Silva F.R., Vettore A.L., Arruda P., Azeredo Espin A.M. (2004). The mitochondrial genome of the blowfly *Chrysomya chloropyga* (diptera: Calliphoridae). Gene.

[76] Sharma D., Issac B., Raghava G.P., Ramaswamy R. (2004). Spectral repeat finder (SRF): Identification of repetitive sequences using fourier transformation. Bioinformatics.

[77] Kurtz S., Choudhuri J.V., Ohlebusch E., Schleiermacher C., Stoye J., Giegerich R. (2001). Reputer: The manifold applications of repeat analysis on a genomic scale. Nucleic Acids Res..

[78] Sharp P.M., Li W.H. (1987). The codon adaptation inde—A measure of directional synonymous codon usage bias, and its potential applications. Nucleic Acids Res..

[79] Stamatakis A. (2014). Raxml version 8: A tool for phylogenetic analysis and post-analysis of large phylogenies. Bioinformatics.

[80] Ronquist F., Huelsenbeck J.P. (2003). Mrbayes 3: Bayesian phylogenetic inference under mixed models. Bioinformatics.

[81] He Z., Zhang H., Gao S., Lercher M.J., Chen W.H., Hu S. (2016). Evolview v2: An online visualization and management tool for customized and annotated phylogenetic trees. Nucleic Acids Res..

[82] Zhang H., Gao S., Lercher M.J., Hu S., Chen W.H. (2012). Evolview, an online tool for visualizing, annotating and managing phylogenetic trees. Nucleic Acids Res..

[83] Kumar S., Stecher G., Suleski M., Hedges S.B. (2017). Timetree: A resource for timelines, timetrees, and divergence times. Mol. Biol. Evol..

