# Supplementary material for: Characterization of the Complete Mitochondrial Genome Sequences of Three Croakers (Perciformes, Sciaenidae) and Novel Insights into the Phylogenetics
Source: Int J Mol Sci. 2018 Jun 12;19(6):1741. doi: 10.3390/ijms19061741 (PMC6032254; doi:10.3390/ijms19061741)
Supplement: Supplementary file 1 [file ijms-19-01741-s001.pdf]

Table S1. Codon Number, Frequency and RSCU (Relative Synonymous Codon Usage) of mitochondrial genomes for Protonibea diacanthus.

Amino acid	Codon	Number	Frequency(%)	RSCU
Ala	GCC	170	4.4702	1.9048
Ala	GCA	115	3.0239	1.2885
Ala	GCT	58	1.5251	0.6499
Ala	GCG	14	0.3681	0.1569
Arg	CGA	46	1.2096	2.3000
Arg	CGC	22	0.5785	1.1000
Arg	CGG	6	0.1578	0.3000
Arg	CGT	6	0.1578	0.3000
Asn	AAC	88	2.3140	1.4915
Asn	AAT	30	0.7889	0.5085
Asp	GAC	62	1.6303	1.6104
Asp	GAT	15	0.3944	0.3896
Cys	TGC	20	0.5259	1.5385
Cys	TGT	6	0.1578	0.4615
Gln	CAA	79	2.0773	1.7363
Gln	CAG	12	0.3155	0.2637
Glu	GAA	73	1.9195	1.4314
Glu	GAG	29	0.7626	0.5686
Gly	GGC	106	2.7873	1.7890
Gly	GGA	62	1.6303	1.0464
Gly	GGG	37	0.9729	0.6245
Gly	GGT	32	0.8414	0.5401
His	CAC	77	2.0247	1.4528
His	CAT	29	0.7626	0.5472
Ile	ATC	147	3.8654	1.0352
Ile	ATT	137	3.6024	0.9648
Leu	CTC	215	5.6534	1.9225
Leu	CTA	180	4.7331	1.6095
Leu	CTT	134	3.5235	1.1982
Leu	TTA	74	1.9458	0.6617
Leu	CTG	48	1.2622	0.4292
Leu	TTG	20	0.5259	0.1788
Lys	AAA	69	1.8144	1.7692
Lys	AAG	9	0.2367	0.2308
Met	ATA	88	2.3140	1.2754
Met	ATG	50	1.3148	0.7246
Phe	TTC	151	3.9705	1.2227
Phe	TTT	96	2.5243	0.7773

Pro	CCC	123	3.2343	2.1674
Pro	CCA	51	1.3410	0.8987
Pro	CCT	41	1.0781	0.7225
Pro	CCG	12	0.3155	0.2115
Ser	TCC	79	2.0773	2.1161
Ser	TCA	61	1.6040	1.6339
Ser	AGC	34	0.8940	0.9107
Ser	TCT	28	0.7363	0.7500
Ser	AGT	14	0.3681	0.3750
Ser	TCG	8	0.2104	0.2143
Stp	TAA	4	0.1052	2.0000
Stp	AGA	2	0.0526	1.0000
Stp	TAG	2	0.0526	1.0000
Stp	AGG	0	0.0000	0.0000
Thr	ACC	136	3.5761	1.8133
Thr	ACA	103	2.7084	1.3733
Thr	ACT	51	1.3410	0.6800
Thr	ACG	10	0.2630	0.1333
Trp	TGA	93	2.4454	1.5630
Trp	TGG	26	0.6837	0.4370
Tyr	TAC	81	2.1299	1.3729
Tyr	TAT	37	0.9729	0.6271
Val	GTA	61	1.6040	1.2513
Val	GTC	60	1.5777	1.2308
Val	GTT	50	1.3148	1.0256
Val	GTG	24	0.6311	0.4923

Tab. S2 Pairwise evolutionary rates of the Argyrosomus amoyensis (AA), Nibea coibor (NC), and Protonibea diacanthus (PD) mitochondrial genomes

	**ATP6**	**ATP8**	**COX1**	**COX2**	**COX3**	**CYTB**	**ND1**	**ND2**	**ND3**	**ND4**	**ND4L**	**ND5**	**ND6**
NC-AA	0.0353	0.1209	0.0175	0.0265	0.0138	0.0190	0.0375	0.0965	0.0725	0.0578	0.0802	0.1926	0.1162
NC-PD	0.0223	0.1366	0.0143	0.0414	0.0226	0.0272	0.0426	0.0834	0.0728	0.0654	0.0448	0.2714	0.1328
AA-PD	0.0417	0.0244	0.0100	0.0305	0.0104	0.0288	0.0297	0.0811	0.0692	0.0379	0.0258	0.2472	0.1162

Table S3. 204 species and their taxonomy of Series Eupercaria for the phylogenetic tree

No.	ID	Species	Order	Family	Genus
1	NC_009830	*Acanthurus leucosternon*	Acanthuriformes	Acanthuridae	*Acanthurus*
2	NC_009853	*Naso lopezi*			*Naso*
3	NC_029237	*Paracanthurus hepatus*			*Paracanthurus*
4	NC_009874	*Zebrasoma flavescens*			*Zebrasoma*
5	NC_009851	*Luvarus imperialis*		Luvaridae	*Luvarus*
6	NC_009852	*Zanclus cornutus*		Zanclidae	*Zanclus*
7	NC_015984	*Lepomis macrochirus*	Centrarchiformes	Centrarchidae	*Lepomis*
8	NC_008106	*Micropterus salmoides*			*Micropterus*
9	NC_028298	*Pomoxis nigromaculatus*			*Pomoxis*
10	NC_024858	*Cheilodactylus zonatus*		Cheilodactylidae	*Cheilodactylus*
11	NC_013181	*Enoplosus armatus*		Enoplosidae	*Enoplosus*
12	NC_013142	*Kuhlia mugil*		Kuhliidae	*Kuhlia*
13	NC_013137	*Girella punctata*		Kyphosidae	*Girella*
14	NC_013138	*Kyphosus cinerascens*			*Kyphosus*
15	NC_013139	*Labracoglossa argenteiventris*			*Labracoglossa*
16	NC_013182	*Microcanthus strigatus*			*Microcanthus*
17	NC_013140	*Scorpis lineolata*			*Scorpis*
18	NC_024853	*Tilodon sexfasciatum*			*Tilodon*
19	NC_010968	*Oplegnathus fasciatus*		Oplegnathidae	*Oplegnathus*
20	NC_024850	*Percalates novemaculeata*		Percalatidae	*Percalates*
21	NC_024847	*Bostockia porosa*		Percichthyidae	*Bostockia*
22	NC_024436	*Gadopsis marmoratus*			*Gadopsis*
23	NC_023807	*Maccullochella peelii*			*Maccullochella*
24	NC_023383	*Macquaria australasica*			*Macquaria*
25	NC_015542	*Nannoperca australis*			*Nannoperca*
26	NC_024846	*Percilia irwini*			*Percilia*
27	NC_009868	*Coreoperca kawamebari*		Sinipercidae	*Coreoperca*
28	NC_015815	*Siniperca scherzeri*			*Siniperca*
29	NC_024854	*Bidyanus bidyanus*		Terapontidae	*Bidyanus*
30	NC_013141	*Rhynchopelates oxyrhynchus*			*Rhynchopelates*
31	NC_027171	*Scortum barcoo*			*Scortum*
32	NC_027281	*Terapon jarbua*			*Terapon*
33	NC_009870	*Chaetodon auripes*	Chaetodontiformes	Chaetodontidae	*Chaetodon*
34	NC_025953	*Chelmon rostratus*			*Chelmon*
35	NC_009871	*Heniochus diphreutes*			*Heniochus*
36	NC_027733	*Ephippus orbis*	Ephippiformes	Ephippidae	*Ephippus*
37	NC_013136	*Platax orbicularis*			*Platax*
38	KM257863	*Argyrosomus amoyensis*	Eupercaria	Sciaenidae	*Argyrosomus*
39	NC_017610	*Argyrosomus japonicus*			

40	NC_018347	*Bahaba taipingensis*			*Bahaba*
41	NC_016987	*Chrysochir aureus*			*Chrysochir*
42	NC_014263	*Collichthys niveatus*			*Collichthys*
43	NC_017606	*Dendrophysa russelii*			*Dendrophysa*
44	NC_021130	*Johnius grypotus*			*Johnius*
45	NC_011710	*Larimichthys crocea*			*Larimichthys*
46	NC_014351	*Miichthys miiuy*			*Miichthys*
47	KM233452	*Nibea coibor*			*Nibea*
48	NC_015205	*Nibea albiflora*			
49	NC_015202	*Pennahia argentata*			*Pennahia*
50	KM257722	*Protonibea diacanthus*			*Protonibea*
51	NC_016867	*Sciaenops ocellatus*			*Sciaenops*
52	NC_028423	*Bodianus oxycephalus*	Labriformes	Labridae	*Bodianus*
53	NC_013842	*Cheilinus undulatus*			*Cheilinus*
54	NC_006355	*Chlorurus sordidus*			*Chlorurus*
55	NC_025771	*Choerodon schoenleinii*			*Choerodon*
56	NC_009066	*Halichoeres melanurus*			*Halichoeres*
57	NC_028289	*Macropharyngodon negrosensis*			*Macropharyngodon*
58	NC_009459	*Parajulis poecilepterus*			*Parajulis*
59	NC_009067	*Pseudolabrus sieboldi*			*Pseudolabrus*
60	NC_010205	*Pteragogus flagellifer*			*Pteragogus*
61	NC_011343	*Scarus rubroviolaceus*			*Scarus*
62	NC_009061	*Olisthops cyanomelas*		Odacidae	*Olisthops*
63	NC_030259	*Datnioides undecimradiatus*	Lobotiformes	Datnioididae	*Datnioides*
64	NC_026233	*Lobotes surinamensis*		Lobotidae	*Lobotes*
65	NC_004383	*Caulophryne jordani*	Lophiiformes	Caulophrynidae	*Caulophryne*
66	NC_013882	*Ceratias uranoscopus*		Ceratiidae	*Ceratias*
67	NC_013880	*Cryptopsaras couesii*			*Cryptopsaras*
68	NC_004381	*Chaunax abei*		Chaunacidae	*Chaunax*
69	NC_013869	*Bufoceratias thele*		Diceratiidae	*Bufoceratias*
70	NC_013870	*Diceratias pileatus*			*Diceratias*
71	NC_013885	*Gigantactis vanhoeffeni*		Gigantactinidae	*Gigantactis*
72	NC_013863	*Rhynchactis macrothrix*			*Rhynchactis*
73	NC_013867	*Himantolophus albinares*		Himantolophidae	*Himantolophus*
74	NC_013865	*Haplophryne mollis*		Linophrynidae	*Haplophryne*
75	NC_013872	*Lophiodes caulinaris*		Lophiidae	*Lophiodes*
76	NC_008125	*Lophiomus setigerus*			*Lophiomus*
77	NC_004380	*Lophius americanus*			*Lophius*
78	NC_013873	*Sladenia gardineri*			*Sladenia*
79	NC_004384	*Melanocetus murrayi*		Melanocetidae	*Melanocetus*
80	NC_013864	*Neoceratias spinifer*		Neoceratiidae	*Neoceratias*

81	NC_013886	*Coelophrys brevicaudata*		Ogcocephalidae	*Coelophrys*
82	NC_013871	*Oneirodes thompsoni*		Oneirodidae	*Oneirodes*
83	NC_013875	*Thaumatichthys pagidostomus*		Thaumatichthyidae	*Thaumatichthys*
84	NC_009867	*Doederleinia berycoides*	Pempheriformes	Acropomatidae	*Doederleinia*
85	NC_028165	*Banjos banjos*		Banjosidae	*Banjos*
86	NC_024852	*Howella brodiei*		Howellidae	*Howella*
87	NC_018045	*Lateolabrax maculatus*		Lateolabracidae	*Lateolabrax*
88	NC_015786	*Histiopterus typus*		Pentacerotidae	*Histiopterus*
89	NC_021758	*Pentaceros japonicus*			*Pentaceros*
90	NC_020016	*Pseudopentaceros richardsoni*			*Pseudopentaceros*
91	NC_027600	*Ulcina olrikii*	Perciformes	Agonidae	*Ulcina*
92	NC_009773	*Anarhichas lupus*		Anarhichadidae	*Anarhichas*
93	NC_018119	*Anoplopoma fimbria*		Anoplopomatidae	*Anoplopoma*
94	NC_026889	*Erilepis zonifer*			*Erilepis*
95	NC_011569	*Aulichthys japonicus*		Aulorhynchidae	*Aulichthys*
96	NC_010268	*Aulorhynchus flavidus*			*Aulorhynchus*
97	NC_026578	*Parachaenichthys charcoti*		Bathydraconidae	*Parachaenichthys*
98	NC_015654	*Chaenocephalus aceratus*		Channichthyidae	*Chaenocephalus*
99	NC_018340	*Champsocephalus gunnari*			*Champsocephalus*
100	NC_029737	*Chionodraco hamatus*			*Chionodraco*
101	NC_013828	*Clinocottus analis*		Cottidae	*Clinocottus*
102	NC_004404	*Cottus reinii*			*Cottus*
103	NC_022147	*Enophrys diceraus*			*Enophrys*
104	NC_027587	*Icelus spatula*			*Icelus*
105	NC_022181	*Mesocottus haitej*			*Mesocottus*
106	NC_018770	*Trachidermus fasciatus*			*Trachidermus*
107	NC_008129	*Aptocyclus ventricosus*		Cyclopteridae	*Aptocyclus*
108	NC_011580	*Apeltes quadracus*		Gasterosteidae	*Apeltes*
109	NC_011577	*Culaea inconstans*			*Culaea*
110	NC_011570	*Gasterosteus wheatlandi*			*Gasterosteus*
111	NC_011571	*Pungitius pungitius*			*Pungitius*
112	NC_011582	*Spinachia spinachia*			*Spinachia*
113	NC_021459	*Hexagrammos agrammus*		Hexagrammidae	*Hexagrammos*
114	NC_026887	*Ophiodon elongatus*			*Ophiodon*
115	NC_023129	*Pleurogrammus azonus*			*Pleurogrammus*
116	NC_004400	*Hypoptychus dybowskii*		Hypoptychidae	*Hypoptychus*
117	NC_018135	*Dissostichus eleginoides*		Nototheniidae	*Dissostichus*
118	NC_015653	*Notothenia coriiceps*			*Notothenia*
119	NC_030320	*Pagothenia borchgrevinki*			*Pagothenia*
120	NC_015652	*Pleuragramma antarctica*			*Pleuragramma*
121	NC_005254	*Etheostoma radiosum*		Percidae	*Etheostoma*
122	NC_025785	*Gymnocephalus cernua*			*Gymnocephalus*

123	NC_019572	*Perca flavescens*			*Perca*
124	NC_008111	*Percina macrolepida*			*Percina*
125	NC_021444	*Sander canadensis*			*Sander*
126	NC_004403	*Scalicus amiscus*		Peristediidae	*Scalicus*
127	NC_004410	*Pholis crassispina*		Pholidae	*Pholis*
128	NC_022481	*Platycephalus indicus*		Platycephalidae	*Platycephalus*
129	NC_024746	*Pterois miles*		Scorpaenidae	*Pterois*
130	NC_027735	*Scorpaenopsis cirrosa*			*Scorpaenopsis*
131	NC_003195	*Helicolenus hilgendorfi*		Sebastidae	*Helicolenus*
132	NC_005450	*Sebastes schlegelii*			*Sebastes*
133	NC_013812	*Sebastiscus marmoratus*			*Sebastiscus*
134	NC_022141	*Aethaloperca rogaa*		Serranidae	*Aethaloperca*
135	NC_012709	*Anyperodon leucogrammicus*			*Anyperodon*
136	NC_021134	*Cephalopholis boenak*			*Cephalopholis*
137	NC_021614	*Cromileptes altivelis*			*Cromileptes*
138	NC_026897	*Diploprion bifasciatum*			*Diploprion*
139	NC_011111	*Epinephelus coioides*			*Epinephelus*
140	NC_024108	*Grammistes sexlineatus*			*Grammistes*
141	NC_013832	*Hypoplectrus gemma*			*Hypoplectrus*
142	NC_013829	*Hyporthodus septemfasciatus*			*Hyporthodus*
143	NC_008449	*Plectropomus leopardus*			*Plectropomus*
144	NC_028286	*Pseudanthias dispar*			*Pseudanthias*
145	NC_022140	*Triso dermopterus*			*Triso*
146	NC_022138	*Variola louti*			*Variola*
147	NC_028022	*Chirolophis japonicus*		Stichaeidae	*Chirolophis*
148	NC_027588	*Leptoclinus maculatus*			*Leptoclinus*
149	NC_026989	*Synanceia verrucosa*		Synanceiidae	*Synanceia*
150	NC_002812	*Arctoscopus japonicus*		Trichodontidae	*Arctoscopus*
151	NC_004409	*Lycodes toyamensis*		Zoarcidae	*Lycodes*
152	NC_009854	*Spicara maena*	Spariformes	Centracanthidae	*Spicara*
153	NC_009855	*Lethrinus obsoletus*		Lethrinidae	*Lethrinus*
154	NC_010957	*Monotaxis grandoculis*			*Monotaxis*
155	NC_010977	*Acanthopagrus latus*		Sparidae	*Acanthopagrus*
156	NC_029479	*Dentex tumifrons*			*Dentex*
157	NC_009502	*Pagellus bogaraveo*			*Pagellus*
158	NC_003196	*Pagrus major*			*Pagrus*
159	NC_008616	*Parargyrops edita*			*Parargyrops*
160	NC_025301	*Rhabdosargus sarba*			*Rhabdosargus*
161	NC_024236	*Sparus aurata*			*Sparus*
162	NC_011319	*Anoplocapros lenticularis*	Tetraodontiformes	Aracanidae	*Anoplocapros*
163	NC_009864	*Kentrocapros aculeatus*			*Kentrocapros*
164	NC_011943	*Abalistes stellaris*		Balistidae	*Abalistes*

165	NC_011946	*Balistapus undulatus*			*Balistapus*
166	NC_011948	*Balistes vetula*			*Balistes*
167	NC_011934	*Balistoides conspicillum*			*Balistoides*
168	NC_011935	*Canthidermis maculata*			*Canthidermis*
169	NC_011937	*Melichthys vidua*			*Melichthys*
170	NC_011938	*Odonus niger*			*Odonus*
171	NC_011939	*Pseudobalistes flavimarginatus*			*Pseudobalistes*
172	NC_011941	*Rhinecanthus aculeatus*			*Rhinecanthus*
173	NC_004416	*Sufflamen fraenatum*			*Sufflamen*
174	NC_011945	*Xanthichthys auromarginatus*			*Xanthichthys*
175	NC_011321	*Xenobalistes tumidipectoris*			*Xenobalistes*
176	NC_005837	*Masturus lanceolatus*		Molidae	*Masturus*
177	NC_005836	*Mola mola*			*Mola*
178	NC_007887	*Ranzania laevis*			*Ranzania*
179	NC_011947	*Acanthaluteres brownii*		Monacanthidae	*Acanthaluteres*
180	NC_011950	*Acreichthys tomentosus*			*Acreichthys*
181	NC_011323	*Aluterus scriptus*			*Aluterus*
182	NC_011952	*Amanses scopas*			*Amanses*
183	NC_011940	*Brachaluteres ulvarum*			*Brachaluteres*
184	NC_011325	*Cantherhines pardalis*			*Cantherhines*
185	NC_011951	*Chaetodermis penicilligerus*			*Chaetodermis*
186	NC_011953	*Eubalichthys mosaicus*			*Eubalichthys*
187	NC_011956	*Meuschenia hippocrepis*			*Meuschenia*
188	NC_011925	*Monacanthus chinensis*			*Monacanthus*
189	NC_011921	*Nelusetta ayraudi*			*Nelusetta*
190	NC_011927	*Oxymonacanthus longirostris*			*Oxymonacanthus*
191	NC_011929	*Paraluteres prionurus*			*Paraluteres*
192	NC_011931	*Paramonacanthus choirocephalus*			*Paramonacanthus*
193	NC_011932	*Pervagor janthinosoma*			*Pervagor*
194	NC_011955	*Pseudalutarius nasicornis*			*Pseudalutarius*
195	NC_011933	*Pseudomonacanthus peroni*			*Pseudomonacanthus*
196	NC_011924	*Rudarius ercodes*			*Rudarius*
197	NC_011926	*Scobinichthys granulatus*			*Scobinichthys*
198	NC_003177	*Stephanolepis cirrhifer*			*Stephanolepis*
199	NC_011327	*Thamnaconus modestus*			*Thamnaconus*
200	NC_011330	*Lactoria diaphana*		Ostraciidae	*Lactoria*
201	NC_009865	*Ostracion immaculatus*			*Ostracion*
202	NC_009859	*Triodon macropterus*		Triodontidae	*Triodon*
203	NC_018037	*Cyprinus carpio haematopterus*	Cypriniformes	Cyprinidae	*Cyprinus*
204	NC_002333	*Danio rerio*			*Danio*

Note: The lineages of the species No. 1-202 are Eukaryota, Metazoa, Chordata, Craniata, Vertebrata, Euteleostomi, Actinopterygii, Neopterygii, Teleostei, Neoteleostei, Acanthomorphata and Eupercaria. And the lineages of the species No. 203-204 are Eukaryota, Metazoa, Chordata, Craniata, Vertebrata, Euteleostomi, Actinopterygii, Neopterygii, Teleostei and Ostariophysi.
